# Effect of Lifelong Exposure to Dietary Plant and Marine Sources of *n*-3 Polyunsaturated Fatty Acids on Morphologic and Gene Expression Biomarkers of Intestinal Health in Early Life

**DOI:** 10.3390/nu16050719

**Published:** 2024-03-01

**Authors:** Julianna E. Acosta, Jessie L. Burns, Lyn M. Hillyer, Kelsey Van, Elaina B. K. Brendel, Camille Law, David W. L. Ma, Jennifer M. Monk

**Affiliations:** 1 Department of Human Health and Nutritional Sciences, University of Guelph, Guelph, ON N1G 2W1, Canada; acosataj@uoguelph.ca (J.E.A.); lhillyer@uoguelph.ca (L.M.H.); kvan@uoguelph.ca (K.V.); ebrendel@uoguelph.ca (E.B.K.B.); claw02@uoguelph.ca (C.L.); davidma@uoguelph.ca (D.W.L.M.); 2 Department of Health Sciences, Carleton University, Ottawa, ON K1S 5B6, Canada; jessie.burns@carleton.ca

**Keywords:** *n*-3 polyunsaturated fatty acids, early life, intestinal morphology, intestinal health, crypt length, goblet cells, fish oil, flaxseed oil

## Abstract

Altered intestinal health is also associated with the incidence and severity of many chronic inflammatory conditions, which could be attenuated via dietary *n*-3 PUFA interventions. However, little is known about the effect of lifelong exposure to *n*-3 PUFA from plant and marine sources (beginning in utero via the maternal diet) on early life biomarkers of intestinal health. Harems of C57Bl/6 mice were randomly assigned to one of three isocaloric AIN-93G modified diets differing in their fat sources consisting of the following: (i) 10% safflower oil (SO, enriched in *n*-6 PUFA), (ii) 3% flaxseed oil + 7% safflower oil (FX, plant-based *n*-3 PUFA-enriched diet), or (iii) 3% menhaden fish oil + 7% safflower oil (MO, marine-based *n*-3 PUFA-enriched diet). Mothers remained on these diets throughout pregnancy and offspring (n = 14/diet) continued on the same parental diet until termination at 3 weeks of age. In ileum, villi:crypt length ratios were increased in both the FX and MO dietary groups compared to SO (*p* < 0.05). Ileum mRNA expression of critical intestinal health biomarkers was increased by both *n*-3 PUFA-enriched diets including *Relmβ* and *REG3γ* compared to SO (*p* < 0.05), whereas only the FX diet increased mRNA expression of *TFF3* and *Muc2* (*p* < 0.05) and only the MO diet increased mRNA expression of *ZO-1* (*p* < 0.05). In the proximal colon, both the FX and MO diets increased crypt lengths compared to SO (*p* < 0.05), whereas only the MO diet increased goblet cell numbers compared to SO (*p* < 0.05). Further, the MO diet increased proximal colon mRNA expression of *Relmβ* and *REG3γ* (*p* < 0.05) and both MO and FX increased mRNA expression of *Muc2* compared to SO (*p* < 0.05). Collectively, these results demonstrate that lifelong exposure to dietary *n*-3 PUFA, beginning in utero, from both plant and marine sources, can support intestinal health development in early life. The differential effects between plant and marine sources warrants further investigation for optimizing health.

## 1. Introduction

The gastrointestinal (GI) microenvironment comprises both the microbiome and the host intestinal defense elements that co-exist, and combined, can profoundly influence host health and physiological function both locally (i.e., within the GI) and systemically (i.e., extra-intestinally) [1]. The mucosa (combined epithelium and lamina propria) of the small intestine and colon structure is the critical tissue at the interface between the microbiome and the host. The contributions of the host toward maintaining balance within the GI microenvironment include critical defense elements and/or physical barriers that protect the mucosa, including (i) the secreted mucus layer/barrier, (ii) the intestinal cellular barrier (i.e., the epithelium), and (iii) an immunological barrier composed of an aggregation of innate and adaptive immune cells with the mucosa (predominantly in the lamina propria) [2,3,4]. Importantly, the mucus layer is composed of mucins secreted by goblet cells that act as a physical barrier between the microbiome and the cellular barrier [5,6]. Additionally, various antimicrobial peptides and proteins are secreted into the mucus layer by the cells within the intestinal epithelium to further fortify this defense element and maintain physical separation between the microbiota and the host [4,7,8]. The intestinal barrier, extending throughout the small intestine and colon regions of the gastrointestinal tract, comprises a single-cell layer that separates the luminal contents from accessing the submucosal layers and the ability to reach the circulatory system and gain access to extra-intestinal tissues [4,7]. The intestinal barrier is predominantly composed of epithelial cells but also contains mucus-secreting goblet cells, enteroendocrine cells, Paneth cells, and stem cells [2,4,7]. Therefore, capacity to sustain these critical elements involved in intestinal barrier function and defense represent important biomarkers of intestinal health. Reduced expression and/or function in GI barrier defense elements can lead to intestinal epithelial barrier dysfunction characterized by increased barrier permeability (i.e., leaky gut) and the translocation of luminal elements (pathogens, bacteria, bacteria cell wall components (e.g., lipopolysaccharide), or bacterial-derived metabolites, pro-inflammatory substances, toxins, antigens, etc.) across the epithelial barrier that can adversely impact host physiological function including triggering or exacerbating host inflammatory responses and metabolic dysfunction [3,4,9]. Therefore, increased intestinal permeability is a common element in many chronic diseases including, but not limited to, inflammatory bowel disease (IBD) and other intestinal disorders, obesity, liver diseases (e.g., non-alcoholic fatty liver disease and non-alcoholic steatohepatitis), type 1 diabetes, and cardiovascular disease [2,4,7,8,9,10]. Therefore, identifying interventions that promote intestinal health and defenses while reducing epithelial barrier permeability are needed [2,9].

Dietary interventions represent a critical and non-invasive way to alter not only the microbiota composition but also affect host intestinal permeability and promote intestinal barrier defensive function [3,4,10]. Therefore, many studies emphasize the barrier integrity promoting effects of microbiota-accessible carbohydrates and the production of short-chain fatty acids and various polyphenolic compounds, as reviewed in [3,4]. With respect to dietary lipids, there is considerable research on the adverse effects of a high fat diet (60% fat as kcal, predominantly comprising saturated fatty acids) on intestinal epithelial barrier integrity and function, as reviewed in [3,4]. This highlights, in part, the relevance of the high-fat diet model to recapitulate the critical features of human obesity in the intestinal microenvironment and the adipose tissue in a diet-induced rodent model [11,12,13,14,15]. The addition of long-chain *n*-3 polyunsaturated fatty acids (PUFA) eicosapentaenoic acid (20:5 *n*-3, EPA) and docosahexaenoic acid (22:6 *n*-3, DHA) to a high-fat diet at 5.3% kcal from menhaden fish oil has been shown to not only improve the adipose tissue obese inflammatory phenotype, but to also increase ileum and colon gene expression of critical biomarkers of intestinal health compared to obese (high-fat diet-fed) controls [11]. Thus, in addition to modifying the microbiota composition, *n*-3 PUFA supplementation also increased expression of apical junctional complex components that promote intestinal epithelial barrier integrity, mucins, and antimicrobial proteins [11]. Therefore, different types of dietary fatty acids, apart from saturated fatty acids, can also impact intestinal health. Furthermore, in epithelial cell culture models, *n*-3 PUFA including α-linolenic acid (ALA, C18:3 *n*-3) from dietary plant sources and marine-derived EPA and DHA were shown to impact intestinal permeability to a similar degree as the *n*-6 PUFA linoleic acid (LA, C18:2 *n*-6) and arachidonic acid (AA, C20:4 *n*-6) [16,17]. However, little is known about the effects of *n*-3 and *n*-6 PUFA on intestinal health biomarkers in vivo, particularly during the early stages of life when the host intestinal defenses are developing, apart from studies conducted in livestock species [18,19].

Early life intestinal health represents a critical developmental period [20,21,22,23,24] that can impact mucosal infection susceptibility [24,25,26] and/or disease susceptibility later in life. As a part of this connection, PUFAs are necessary for normal growth and development, cellular function, immune response, and may be critical for the prevention of chronic inflammation and many chronic diseases [27]. Lifelong dietary exposure to *n*-3 PUFA, namely in utero (via maternal dietary intake) and continued dietary exposure in the offspring, has been shown to impact mammary gland development and breast cancer severity later in life [28,29,30,31]. However, there is limited knowledge about the effects of plant- versus marine-derived PUFA in other tissues, such as the small intestine and colon. Therefore, in the current study we investigated the effects of lifelong dietary exposure (namely from in utero to weaning at 3 weeks of age) of *n*-3 PUFA as ALA (plant-derived *n*-3 PUFA) and EPA/DHA (marine-derived *n*-3 PUFA) on early life host intestinal health biomarkers.

## 2. Materials and Methods

### 2.1. Mice, Housing, and Diets

Adult C57BL/6 mice were housed in ventilated cages in a temperature- and humidity-controlled animal facility and were exposed to a 12 h light/12 h dark cycle. Mouse harems comprising one male and three females were randomly assigned to one of three isocaloric experimental diets wherein the formulation was modified from the AIN-93G formulation and contained the same amount of fat (10% *w*/*w*) but differed in the sources of fat (Research Diets Inc., New Brunswick, NJ, USA). The compositions of the three experimental diets are shown in Table 1 and included the following: (i) 10% safflower oil (SO, enriched in *n*-6 PUFA), (ii) 3% flaxseed oil + 7% safflower oil (FX, plant-based *n*-3 PUFA diet), or (iii) 3% menhaden fish oil + 7% safflower oil (MO, marine-based *n*-3 PUFA diet). 

Diet pellets were stored at −20 °C and thawed prior to feeding. Mice were provided with fresh diet pellets every two days. All mice had ad libitum access to food and double-distilled water. Diet fatty acid composition was confirmed with gas chromatography and is shown in Supplemental Materials (Table S1). Mothers remained on their same experimental diets throughout pregnancy, and equal numbers of male and female offspring (n = 14/diet; 7 males and 7 females) were continued on the same parental diet until termination at 3 weeks of age. Once the offspring were terminated, small intestine (ileum) and proximal colon tissue samples were collected and fixed for histology (described below) or snap-frozen in liquid nitrogen and stored at −80 °C to await further analyses. All animal protocols and procedures were performed under Animal Utilization Protocol #4368, which was approved by the Animal Care Committee of the University of Guelph under the governance of the Canadian Council on Animal Care.

### 2.2. Phospholipid Fatty Acid Analysis

Lipids were extracted from ileum via the Folch Method [32] and phospholipid fractions were separated by thin layer chromatography (TLC) as previously described [29]. Briefly, samples were spotted on H-plates (EMD Chemicals, Gibbstown, NJ, USA) to separate phospholipid species. Bands corresponding to lyso-phosphatidylcholine (lyso-PC), sphingomyelin (SM), phosphatidylcholine (PC), phosphatidylserine (PS), phosphatidylinositol (PI), and phosphatidylethanolamine (PE) were collected and methylated with 14% boron trifluoride-methanol (Fisher Scientific, Mississauga, ON, Canada). Fatty acid methyl esters were separated on a DB-FFAP fused-silica capillary column (15 m, 0.1 m film thickness, 0.1 mm i.d.; Agilent, Mississauga, ON, Canada) and quantified on an Agilent 6890 gas chromatograph. Peaks were identified by retention times of fatty acid methyl ester standards (Nu-Chek-Prep, Elysian, MN, USA) using EZchrom Elite version 3.2.1 software. Fatty acid results were calculated as percent composition or ug/0.1 g tissue with 10 µg of heptadecanoic acid (17:0) added to each fraction for the internal standard.

### 2.3. RNA Isolation and qRT-PCR

Ileum and proximal colon tissue was homogenized in the lysis buffer provided in the RNA/Protein Purification Plus Kit (Norgen Biotek Corp., Thorold, ON, Canada) and RNA was isolated following the manufacturer’s instructions. The high-capacity cDNA reverse transcription kit (Applied Biosystems, Foster City, CA, USA) was used to make 2 μg of cDNA. Real-Time PCR was performed using a CFX Real-Time PCR System (Bio-Rad, Mississauga, ON, Canada) as previously described [33]. Primers were designed using the Universal Probe Library Assay Design Center (Roche Applied Sciences, Penzberg, Germany), and validated primer efficiencies were between 90% and 105%. All primer sequences have been published previously [34,35,36,37]. Results were normalized to the housekeeping gene *RPLP0* (*ribosomal protein*, *large*, *P0*) and relative differences in gene expression (expressed in arbitrary units) between treatment groups were determined according to the calculation 2^(40−Ct)^.

### 2.4. Ileum and Colon Morphology

For tissue morphology analyses, proximal colon and ileum tissues were fixed using formalin, embedded in paraffin, cross-sectioned (5 μm), and placed on glass slides. All morphology assessments were made in a blinded manner. Cross-sections were stained with Hematoxylin and Eosin (Millipore-Sigma, Oakville, ON, Canada) for analysis of crypt and villi length. Crypt length measurements in the proximal colon were assessed using approximately 20 fully elongated and intact crypt structures per mouse (n = 14/group) at 40× magnification. Villi length and crypt depth measurements were taken from cross-sections of the ileum using a minimum of 10 fully elongated and intact villi and crypt structures per mouse (n = 12/dietary group) at 40× magnification. The villus length was measured from the baseline to the tip of the villus and the crypt depth was measured from the baseline of the villus to the muscle layer, with villi:crypt length ratios calculated by dividing villus length by crypt depth, as described previously [38]. Ileum and proximal colon cross-sections were also stained with Alcian Blue/Nuclear Fast Red (Millipore-Sigma) to permit quantification of goblet cells per tissue cross-section field of view, with a minimum of 4–5 fields of view per mouse (n = 5–6 mice/dietary group in the ileum and n = 10 mice/dietary group in the proximal colon). Images were collected at 40× magnification. All images of tissue cross-sections were captured using EVOS M7000 Imaging System (Thermo Fisher Scientific, Mississauga, ON, Canada) and analyzed using the National Institute of Health Image J software (version 1.51h).

### 2.5. Statistical Analysis

Gas chromatography data are presented as means ± SD. All other data are expressed as means ± SEM. All data sets were investigated for an effect of sex but there were no statistically significant differences in any outcome. Therefore, data from males and females were pooled together and analyzed using a one-way ANOVA followed by Tukey’s multiple comparison test for post hoc analysis between groups. The Shapiro–Wilk test was used to test for normality. Data were transformed (where applicable) for normality and data that were not normally distributed were analyzed using the Kruskal–Wallis test followed by the Wilcoxon two-sample test. The upper limit of probability for statistical significance was set at *p* ≤ 0.05. Statistical analyses were conducted using the SAS system for windows, version 9.1 (SAS Institute, Cary, NC, USA) and GraphPad Prism version 9.3. (GraphPad Software, Inc., La Jolla, CA, USA).

## 3. Results

### 3.1. Final Body Weight

Weanling mouse final body weights were collected prior to sacrifice. Mice with lifelong exposure to dietary *n*-3 PUFA-enriched diets (MO and FX) exhibited increased final body weights compared to the *n*-6 PUFA-enriched SO dietary group (*p* ≤ 0.05); however, there was no difference between the MO and FX dietary groups (*p* > 0.05). Specifically, final body weight in the FX group (9.55 ± 0.09 g) was increased compared to SO (8.52 ± 0.10 g), which translated into FX mice being 10.8% heavier. The final body weight in MO-fed mice was 9.10 ± 0.56 g, which translated into these mice being 6.4% heavier compared to SO-fed mice.

### 3.2. Ileum Fatty Acid Analysis

**Figure 1:**
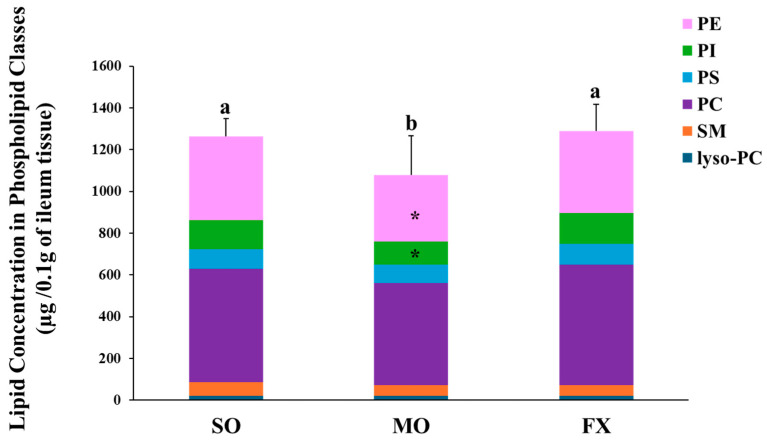
Lipid concentration in phospholipid classes. Bars (mean ± S.D.) represent total amount of phospholipid (µg/0.1 g ileum tissue) found in mouse ileum of each dietary group. Different letters denote significant differences between means, n = 6 per dietary group. Bars are further divided to show the amount of each phospholipid class; lyso-PC (blue), SM (orange), PC (purple), PS (yellow) PI (green), and PE (pink). The asterisk (*) denotes phospholipid classes that were significantly decreased in the MO dietary group (*p* ≤ 0.05). Values for each phospholipid class and PC:PE ratio can be found in Supplementary Materials (Table S2).

Total phospholipid and phospholipids fatty acid composition of lyso-PC, SM, PC, PS, PI, and PE were measured in ileum of mice fed diets enriched in *n*-6 (SO) or *n*-3 PUFA (FX and MO). The lipid concentration in each phospholipid class is shown in Figure 1. Mice fed the MO diet had significantly lower total phospholipid levels than mice fed the SO or FX diets (*p* ≤ 0.05). When examining the contribution of individual phospholipid species to totally phospholipid levels, mice fed the MO diet had significantly lower PI and PE levels when compared to the SO and FX diets (*p* < 0.05), which likely contributed to the decrease in total phospholipid levels. However, this difference in phospholipid-specific levels across dietary groups was not seen for the other phospholipid species, as lyso-PC, SM, PC, and PS levels were the same across all three dietary groups. Further, there was no effect of diet on the PC:PE ratio as the PC:PE ratio was the same across all dietary groups (Table S2).

**Figure 2:**
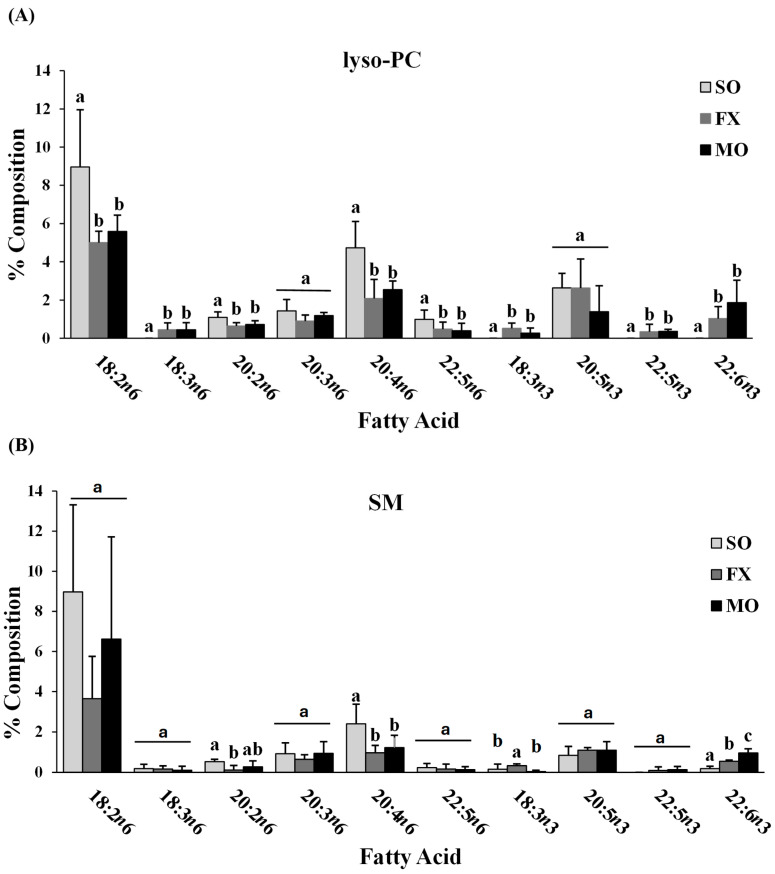

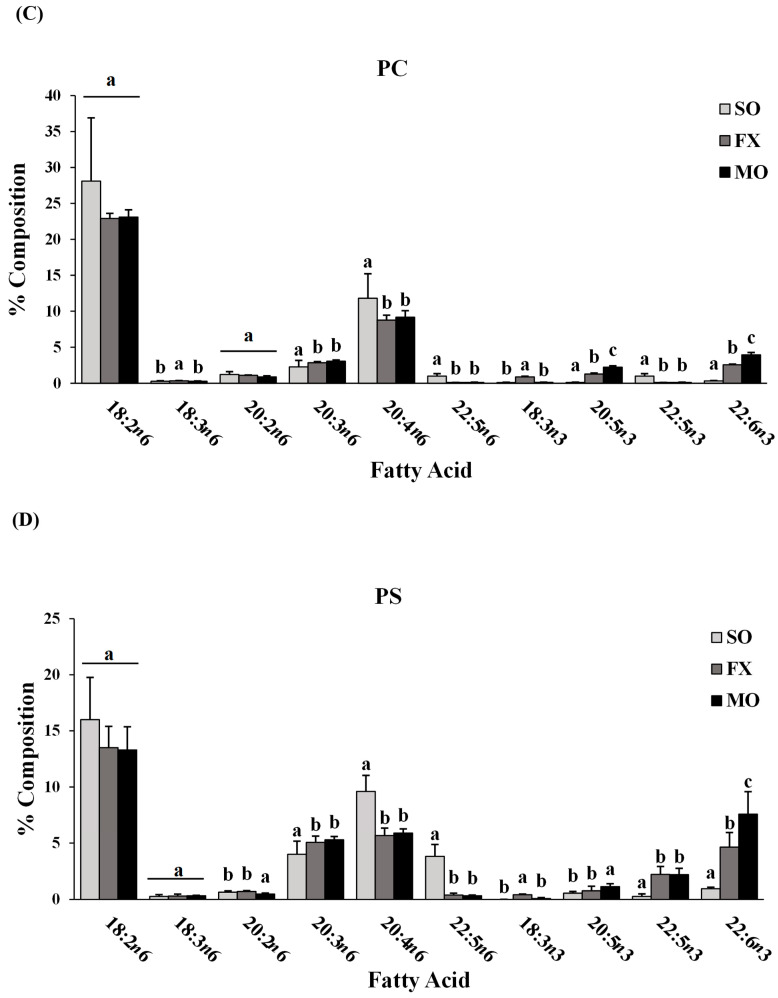

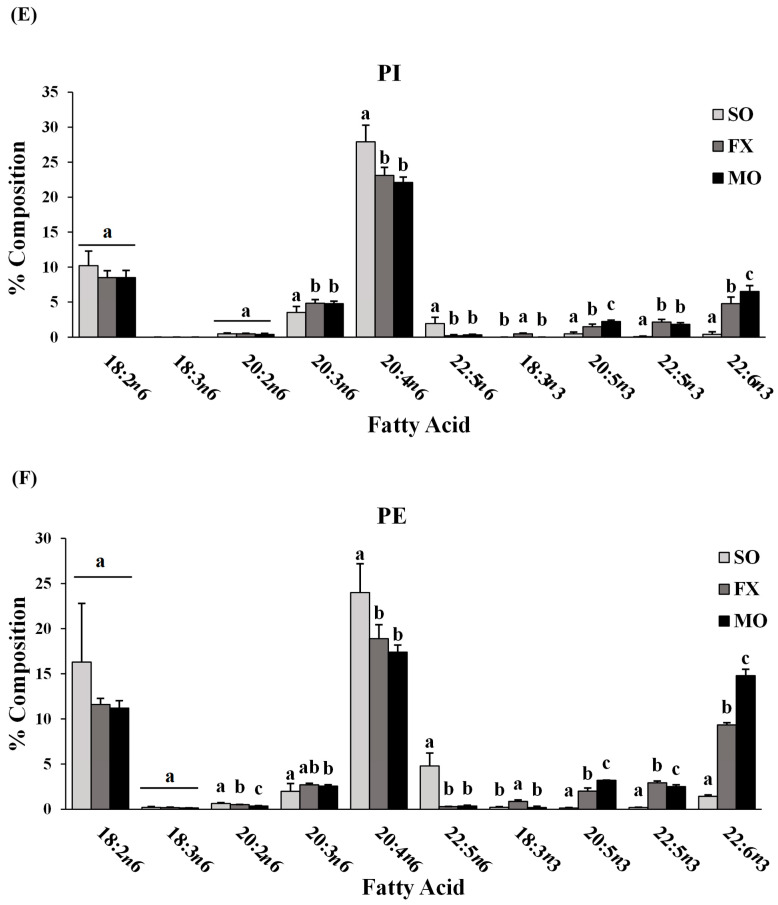
Fatty acid composition. Percent composition of major *n*-6 and *n*-3 PUFA in the lyso-PC (**A**), SM (**B**), PC (**C**), PS (**D**), PI (**E**), and PE (**F**) phospholipid fractions in 3-week-old mouse ileum. Bars represent means + S.D. For each fatty acid, bars not sharing a lowercase letter differ (*p* ≤ 0.05; n = 6/dietary group). Complete fatty acid profiles of each phospholipid fraction can be found in Supplementary Materials (Tables S3–S8, respectively).

Fatty acid composition of each phospholipid species was also determined. All fatty acids detected in each phospholipid species across all dietary groups are shown in Supplemental Materials (lyso-PC in Table S3; SM in Table S4; PC in Table S5; PS in Table S6; PI in Table S7; PE in Table S8). The major *n*-3 and *n*-6 PUFA found in each phospholipid species of mice fed the SO, FX, or MO diets are shown in Figure 2. Within each dietary group and across all phospholipid species, fatty acid data were reflective of dietary PUFA intake. Mice fed the diets high in *n*-3 PUFA (FX and MO) had significantly higher total *n*-3 PUFA composition compared to mice fed the control *n*-6 PUFA diet (SO) across all phospholipid species (*p* ≤ 0.05). In tandem with elevated *n*-3 PUFA, levels of arachidonic acid (AA, 20:4 *n*-6) and docosapentaenoic acid (DPA, 22:5 *n*-6) were significantly lower in the *n*-3 PUFA-fed mice compared to mice fed SO (*p* ≤ 0.05). Mice fed the FX diet (high in α-linolenic acid [ALA, 18:3-*n*3]) had higher ALA incorporation in the SM, PC, PS, PI, and PE phospholipid species when compared to the MO and SO diets (*p* ≤ 0.05), which is reflective of the higher ALA content of the FX diet. Mice fed the MO diet (high in eicosapentaenoic acid [EPA, 20:5*n*3] and docosahexaenoic acid [DHA, 22:6*n*3]) had higher EPA incorporation in the PC, PS, PI, and PE phospholipid species and higher DHA incorporation in the SM, PC, PS, PI, and PE phospholipid species when compared to the FX and SO diets, which is reflective of the higher EPA and DHA contents in the MO diet (*p* ≤ 0.05). Mice fed the SO diet high in *n*-6 PUFA [LA, 18:2*n*6] had significantly higher total *n*-6 PUFA composition compared to mice fed the diets high in *n*-3 PUFA (MO and FX) (*p* ≤ 0.05). As expected, mice fed the SO diet also had significantly higher LA incorporation in the lyso-PC phospholipid species and significantly higher *n*-6/*n*-3 PUFA ratios across all phospholipid species when compared to the MO and FX diets (*p* ≤ 0.05). These phospholipid findings are similar to the findings reported in mouse mammary gland and mammary tumors in other studies utilizing similar dietary compositions [30].

When further examining the fatty acid composition of the phospholipid fractions, the majority of the PE phospholipid fraction was composed of PUFA (compared to MUFA and SFA), with approximately half (52–54%) of all lipid species being PUFA. This, coupled with the higher levels of total *n*-3 PUFA in the PE fraction of mice fed the diets high in *n*-3 PUFA (MO and FX) and higher total *n*-6 PUFA in the PE fraction of mice fed the SO diet, suggests preferential incorporation of dietary PUFA (both *n*-3 and *n*-6 PUFA) into the PE phospholipid species of ileum tissue.

### 3.3. Effect of n-3 PUFA on Biomarkers of Intestinal Health in the Ileum

**Figure 3:**
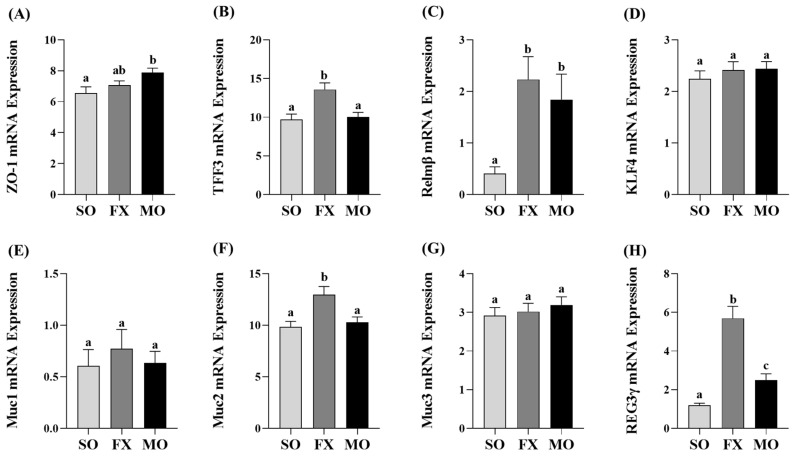
Ileum mRNA expression of intestinal health biomarkers *ZO-1* (**A**), *TFF3* (**B**), *Relmβ* (**C**), *KLF4* (**D**), *Muc1* (**E**), *Muc2* (**F**), *Muc3* (**G**), and *REG3γ* (**H**). Bars represent means ± SEM. Data were analyzed with one-way ANOVA followed by Tukey’s multiple comparison test (n = 10–12/dietary group). Bars not sharing a lowercase letter differ (*p* ≤ 0.05).

Ileum mRNA expression of biomarkers of intestinal health are shown in Figure 3. Zonula occludens (*ZO-1*) mRNA expression was increased in the MO group compared to SO (*p* ≤ 0.05), whereas the FX group exhibited an intermediate expression level that did not differ from either SO or MO (*p* > 0.05; Figure 3A). Expression of trefoil factor 3 (*TFF3*) was significantly increased in the FX group, compared to both SO and MO (*p* ≤ 0.05; Figure 3B). Resistin-like molecule β (*Relmβ*) mRNA expression was significantly increased in both the FX and MO groups compared to SO (*p* ≤ 0.05), but the expression level did not differ between the two *n*-3 PUFA-enriched dietary groups (Figure 3C). Krüppel-like factor 4 (*KLF4*), mucin 1 (*Muc1*), and mucin 3 (*Muc3*) expression did not differ between dietary groups (*p* > 0.05; Figure 3D,E,G), whereas mucin 2 (*Muc2*) expression was increased in the FX group compared to both SO and MO (*p* ≤ 0.05; Figure 3F). Finally, Regenerating family member gamma (*REG3γ*) mRNA expression was increased in both the FX and MO groups compared to SO (*p* ≤ 0.05), wherein the expression level was significantly higher in the FX group (*p* ≤ 0.05; Figure 3H).

**Figure 4:**
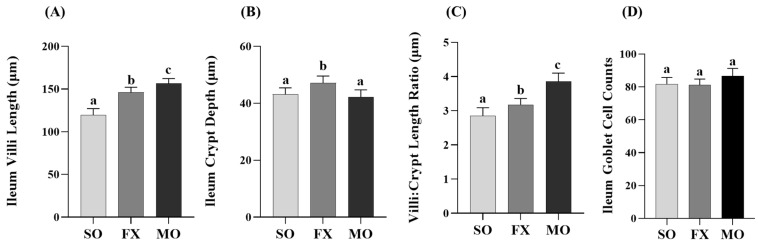
Ileum histomorphology. Bars represent means ± SEM (**A**) villi length, (**B**) crypt depth, (**C**) villi:crypt length ratio, and (**D**) goblet cell counts. Data were analyzed with one-way ANOVA followed by Tukey’s multiple comparison test (n = 12/dietary group for length/depth measurements and n = 10/dietary group for goblet cell analyses). Bars not sharing a lowercase letter differ (*p* ≤ 0.05).

Outcomes from a histological analysis of ileum villi lengths and crypt depths are shown in Figure 4. Villi length was significantly increased in both the MO and FX groups compared to SO (*p* ≤ 0.05), wherein villi in the MO group were significantly longer compared to the FX group (*p* ≤ 0.05; Figure 4A). Conversely, ileum crypt depths were significantly increased in the FX group compared to both SO and MO (*p* ≤ 0.05) and there was no difference in ileum crypt depth between the MO and SO groups (*p* > 0.05; Figure 4B). The aforementioned two morphological measurements were used to assess the villi:crypt length ratio, as shown in Figure 4C. Both *n*-3 PUFA-enriched dietary groups exhibited increased villi:crypt length ratios compared to SO (*p* ≤ 0.05), wherein this ratio was significantly higher in the MO group compared to FX (*p* ≤ 0.05). Ileum goblet cell counts in tissue cross-sections did not differ between dietary groups (*p* > 0.05; Figure 4D).

### 3.4. Effect of n-3 PUFA on Biomarkers of Intestinal Health in the Proximal Colon

**Figure 5:**
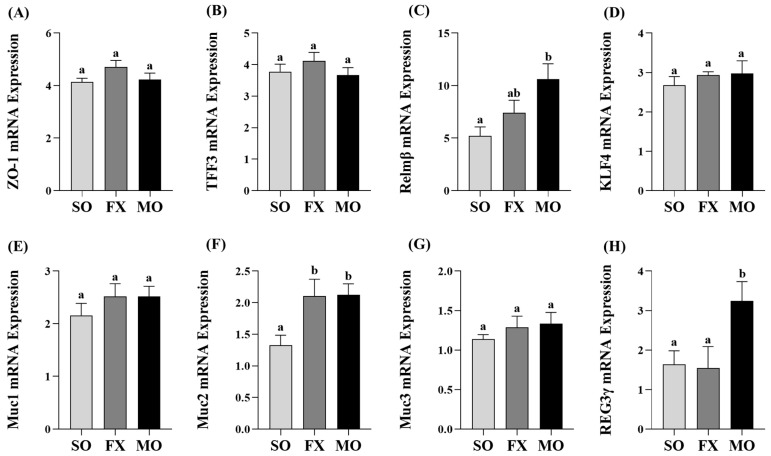
Proximal colon mRNA expression of intestinal health biomarkers *ZO-1* (**A**), *TFF3* (**B**), *Relmβ* (**C**), *KLF4* (**D**), *Muc1* (**E**), *Muc2* (**F**), *Muc3* (**G**), and *REG3γ* (**H**). Bars represent means ± SEM. Data were analyzed with one-way ANOVA followed by Tukey’s multiple comparison test (n = 10–12/dietary group). Bars not sharing a lowercase letter differ (*p* ≤ 0.05).

Proximal colon mRNA expressions of intestinal health biomarkers are shown in Figure 5. The MO group exhibited significantly higher mRNA expression of both *Relmβ* and *REG3γ* compared to both SO and FX (*p* ≤ 0.05; Figure 5C,H, respectively). There was no difference in *Relmβ* and *REG3γ* mRNA expression between the SO and FX groups (*p* > 0.05). Conversely, both the MO and FX groups exhibited significantly higher mRNA expression of *Muc2* compared to SO (*p* ≤ 0.05; Figure 5F); however, the expression level did not differ between the two *n*-3 PUFA-enriched dietary groups (*p* > 0.05). There were no differences in mRNA expression between any dietary groups for other intestinal health biomarkers including *ZO-1*, *TFF3*, *KLF4*, *Muc1*, or *Muc3* (*p* > 0.05; Figure 5).

**Figure 6:**
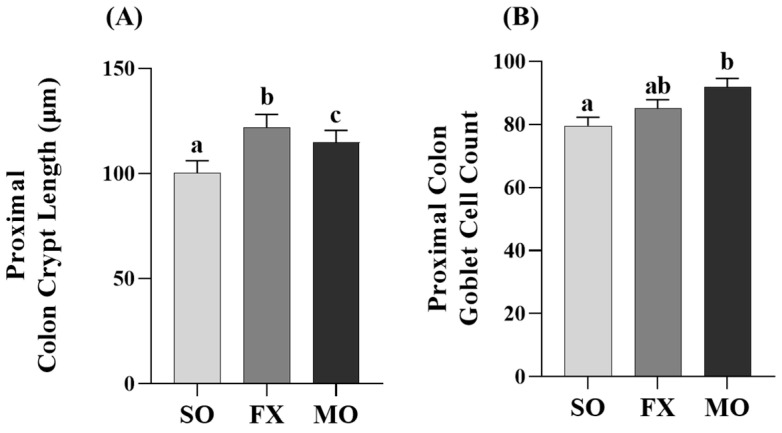
Proximal colon histomorphology. Bars represent means ± SEM. Crypt length (**A**) and goblet cell counts (**B**). Data were analyzed with one-way ANOVA followed by Tukey’s multiple comparison test (n = 12/dietary group for crypt length measurements and n = 10/dietary group for goblet cell analyses). Bars not sharing a lowercase letter differ (*p* ≤ 0.05).

Proximal colon crypt lengths were increased in both the MO and FX groups compared to SO (*p* ≤ 0.05; Figure 6A), wherein crypt lengths were significantly longer in the FX group compared to MO (*p* ≤ 0.05). Analysis of goblet cells within the proximal colon were higher in the MO group compared to SO (*p* ≤ 0.05; Figure 6B); however, there was no difference in goblet cells in the FX group compared to either SO or MO (*p* > 0.05).

## 4. Discussion

This study demonstrates the effects of lifelong (beginning in utero with the maternal diet) exposure to dietary *n*-3 PUFA from either plant (FX diet enriched in ALA) and marine (MO diet enriched in EPA and DHA) sources on critical gene expression and histomorphology biomarkers of intestinal health in early life, specifically in 3-week-old weanling mice. Proper nutrition during critical periods of growth and development may significantly impact an individuals’ short- and long-term health outcomes [39] and establishment of a functional intestinal barrier in early life/weanling stage is critical for mediating resistance to mucosal infection [24,25,26]. As such, lifelong dietary exposure, beginning in utero, has the potential to not only impact fetal development, but may also impact growth, the establishment of intestinal health, and either susceptibility to or severity of disease outcomes developing later in life. However, many of the studies that have assessed the relationship between dietary PUFA exposure and various disease outcomes have not examined the impact of lifelong exposure, as dietary interventions are typically introduced in the post-weaning period [40,41]. The limited studies that have examined the relationship between lifelong dietary exposure to *n*-3 and *n*-6 PUFA and developmental or disease-related outcomes have shown that diet influences the fatty acid composition of extra-intestinal tissues in mice as young as 3 weeks of age [28,29]. Moreover, lifelong exposure to *n*-3 PUFA has been shown to beneficially impact mammary gland development, terminal end bud numbers, and in later life, breast cancer severity [28,29,30,31]. Limited evidence from livestock species suggests a positive benefit on intestinal health [18,19]. Feeding pigs a maternal diet enriched in ALA from linseed oil during pregnancy and lactation did not affect newborn offspring intestinal morphology; however, *n*-3 PUFA impacted intestinal permeability and increased ZO-1 protein expression [19]. Furthermore, broiler chicks fed *n*-3 PUFA during the growth phase post-hatch also exhibited no change in intestinal morphology (apart from decreased distance between villi); however, mRNA expression of Muc2 was increased [18]. Interestingly, increased villi height was positively correlated with Muc2 expression and final body weight [18]. In contrast to the studies in livestock species that showed no effect of *n*-3 PUFA on body weight [18,19], body weights in the current study were modestly increased in both FX-fed and MO-fed mice compared to SO controls. This aligns with previous findings that have shown increased body weights in *n*-3 PUFA-fed mice [28,42]. Early life (i.e., until weanling) establishment of the intestinal microenvironment, which includes contribution from both microbiota and the host that grow in tandem and can affect both early life growth and overall intestinal health [20,21,24,43,44,45]. The transition from suckling to weanling and the consumption of solid food is a critical period for both whole body and intestinal growth, specifically, the development of an intact epithelial and mucus barrier and reduced barrier permeability [20,21,22,23,24]. Furthermore, establishment of intestinal barrier defenses is critical for early life protection from mucosal infections that are associated with diminished intestinal barrier defenses in early life [24,25,26], and thus, identifying dietary approaches that promote early life intestinal health and growth are needed.

The intestinal-health-promoting effects of *n*-3 PUFA supplementation have been studied in the context of established conditions with compromised intestinal health, such as obesity [11], obesity-associated colitis [46], or IBD/experimental colitis studies using long-chain *n*-3 PUFA from marine/fish oil [47,48,49,50] or plant/flaxseed oil [47,51,52,53], with some studies reporting no beneficial effects [54,55,56]. Additionally, there is evidence that *n*-3 PUFA can also promote intestinal healing and restoration of the intestinal epithelial barrier [48,54]. This indicates that the overall effect of *n*-3 PUFA within the intestinal microenvironment can both strengthen intestinal barrier defenses and promote resolution of compromised barrier defenses, but the specific mechanisms in an unchallenged intestinal microenvironment need to be determined. Importantly, the aforementioned studies utilized *n*-3 PUFA as a dietary intervention to attenuate established intestinal disease severity, where *n*-3 PUFA was consumed concurrent with intestinal disease development, and therefore, did not address the significance of utilizing *n*-3 PUFA from a prevention standpoint to improve intestinal health prior to disease onset. In this connection, the intestinal health priming effect of consuming whole ground flaxseed (enriched in ALA but also non-digestible carbohydrates and phenolic compounds) was demonstrated to improve critical biomarkers of intestinal health in healthy unchallenged adult mice [35], which could alter the responsiveness of the intestinal microenvironment upon the initiation of an intestinal-health-compromising condition. Although the effects of whole flaxseed cannot be attributed exclusively to ALA, the results provide support for the potential intestinal health priming effects of *n*-3 PUFA (from either plant or marine sources) that could attenuate disease severity later in life. From this perspective, conditions like obesity and IBD/colitis are associated with compromised intestinal health and increased epithelial barrier permeability, as reviewed in [3,4], and therefore, adequate intakes of dietary *n*-3 PUFA prior to the development of intestinal conditions that compromise intestinal health could attenuate disease severity, although further study is required. Thus, determining the intestinal-health-promoting effects of *n*-3 PUFA through a lifelong exposure model, from a disease prevention standpoint, increases the translational potential of this research.

In the current study, both plant and marine sources of dietary *n*-3 PUFA were shown to modulate critical biomarkers of intestinal health used previously [18,19,35], including intestinal morphology and gene expression of critical barrier defense elements. Changes in intestinal morphology including lengthening of colonic crypts, elongation of intestinal villi, and the villi:crypt length ratio are frequently interpreted to reflect an improvement in intestinal health and in the small intestine to aid in improved digestion and absorption of nutrients [57,58]. In the ileum, both the MO and FX diets improved the morphology of the small intestine including increasing villus length and the villi:crypt length ratio compared to SO (Figure 4), wherein the FX diet significantly increased crypt depth compared to other dietary group. Proximal colon morphology was also affected by the *n*-3 PUFA diets in a manner reflective of improved intestinal health. Both the MO and FX diets increased crypt length measurements and the MO diet increased goblet cell counts, which are the cell types responsible for increased mucin secretion (Figure 6). Relating to this connection, *Muc2* mRNA expression was increased by the FX diet in both the ileum and proximal colon (Figure 3F and Figure 5F), whereas the MO diet increased *Muc2* mRNA expression in only the proximal colon (Figure 5F). In relation to barrier defense, Muc2 is the dominant secreted gel-forming mucin secreted by goblet cells to form the outer mucus layer, and Muc2 knockout mice gradually develop colitis by six months of age [59]. This emphasizes the critical role Muc2 contributes to mucus barrier defense mechanisms within the gastrointestinal tract [60] and highlights how the mucus barrier plays a fundamental role in epithelial barrier function and intestinal health homeostasis within the intestinal mucosa [61]. The FX and MO diets had similar effects in both the ileum and proximal colon on mRNA expression of *REG3γ* and *Relmβ*. Specifically, both *n*-3 PUFA-enriched diets increased ileum expression of *REG3γ* and *Relmβ* (Figure 3C,H); however, only the MO diet increased *REG3γ* and *Relmβ* mRNA expression in the proximal colon (Figure 5C,H, respectively). REG3γ exerts its antimicrobial effects against Gram-positive bacteria, which functions to maintain a physical segregation of the microbiota from the host tissues and limits bacterial colonization of mucosal surfaces [7,62]. Therefore, REG3γ plays a key role in shaping the microbial community structure and regulating host microbial defense responses [7,62,63]. Relmβ is relevant for intestinal health as it functions to promote mucosal barrier integrity via up-regulating mucin secretion to support and maintain the protective mucus barrier, while also exerting immunoregulatory effects that attenuate mucosal damage and inflammation by promoting noninflammatory adaptive responses to intestinal infections [64,65,66,67]. Other diet-specific effects on mRNA expression of intestinal health biomarkers included the increased ileum mRNA expression of *ZO-1* in MO-fed mice (Figure 3A) and the effect of FX in increasing ileum mRNA expression of *TFF3* (Figure 3B). This is functionally relevant as ZO-1 is an intracellular scaffold protein that binds to both the actin cytoskeleton and to the cytoplasmic peripheral membrane proteins found in tight junctions to form strong cross-links [7,68]. ZO-1 contributes to membrane integrity, and although deletion of ZO-1 results in minor increases in barrier permeability, it has recently been shown to contribute to intestinal health by playing an essential role in epithelial cell proliferation and mucosal barrier repair [69,70]. TFF3 is expressed by goblet cells in the small intestine and colon and is usually expressed with Muc2 [71]. Functionally, TFF3 has been shown to function through a paracrine signaling mechanism on nearby epithelial cells to play a key role in both the maintenance and repair of the intestinal mucosa, in part, via increasing epithelial barrier integrity and reducing permeability by modulating tight junction protein expression [71,72,73,74]. There was no effect of diet observed on mRNA expression of (i) *Muc1*, a transmembrane mucin that functions to protect the epithelial barrier [64,75,76], (ii) *Muc3*, an inner mucus layer mucin that regulates epithelial layer integrity and promotes epithelial restitution and wound healing [64,77,78], or (iii) *KLF4*, which initiates goblet cell differentiation and plays a role in the regulation of epithelial barrier homeostasis and morphology [79,80,81]. Collectively, the FX and MO diets improved critical aspects of intestinal health including ileum and proximal colon morphology and gene expression of antimicrobial proteins (*REG3γ*), secreted mucins (*Muc2*), and mediators that play a key role in promoting mucosal barrier integrity (*Relmβ*, *ZO-1*, *TFF3*). Importantly, the majority of intestinal health outcomes that were assessed herein were beneficially affected by both plant-derived (i.e., ALA in the FX diet) and marine-derived *n*-3 PUFA (i.e., EPA and DHA in the MO diet), with only gene expression of a few select outcomes that differed between dietary groups, indicating that both plant and marine sources of *n*-3 PUFA can beneficially modulate early life intestinal health.

## 5. Conclusions

Further study is required to more comprehensively assess other critical elements of intestinal health that were not assessed herein, for example microbiome sequencing, functional measurements of barrier permeability, and protein expression of a more comprehensive assessment of barrier defense elements. Importantly, the current data highlight the proof-of-concept that lifelong *n*-3 PUFA intakes from either plant or marine dietary sources (starting in utero with the maternal diet and continuing through to weaning at 3 weeks of age in the offspring) can improve early life intestinal health biomarkers that could help either prevent or attenuate early life mucosal infections and/or intestinal disease severity in later life. Having determined the beneficial effects of lifelong *n*-3 PUFA intakes on early life intestinal health, it will also be useful to determine the influence of the timing of introducing *n*-3 PUFA into the maternal diet on the development of the intestinal microenvironment in the offspring. Further research is also warranted to better understand the fundamental effects attributed individually to ALA, EPA, and DHA in early life intestinal health.

## Figures and Tables

**Table 1:** Diet composition ^1^.

	SO (D04092701)		MO (D04092703)		FX (D04092711N)	
Macronutrient	g%	kcal%	g%	kcal%	g%	kcal%
Protein	21	20	21	20	21	20
Carbohydrate	60	58	60	58	60	58
Fat	10	22	10	22	10	22
Ingredient	g/kg	kcal	g	kcal	g	kcal
Casein	200	800	200	800	200	800
L-Cystine	3	12	3	12	3	12
Corn starch	337	1347	337	1347	337	1347
Maltodextrin 10	132	528	132	528	132	528
Sucrose	100	400	100	400	100	400
Cellulose, BW200	50	0	50	0	50	0
Safflower oil	97	873	68	611	68	611
Menhaden oil	0	0	29	263	0	0
Flax oil	0	0	0	0	29	263
t-Butylhydroquinone	0.02	0	0.02	0	0.02	0
Mineral Mix S10022 G	35	0	35	0	35	0
Vitamin Mix V10037	10	40	10	40	10	40
Choline bitartrate	2.5	0	2.5	0	2.5	0
Total	966	4000	966	4000	966	4000

^1^ AIN-93G rodent diets were modified to contain 10% safflower oil (SO), 3% menhaden oil + 7% safflower oil (MO), or 3% flaxseed oil + 7% safflower oil (FX).

## Data Availability

Data are contained within the article. The data presented in this study are available upon reasonable request sent to the corresponding author.

## Supplementary Materials

The following supporting information can be downloaded at https://www.mdpi.com/article/10.3390/nu16050719/s1. Table S1: Fatty acid composition of modified AIN-93G rodent diets; Table S2: Lipid concentration (µg/0.1 ileum) in phospholipid classes and PC:PE ratio; Table S3: Percent composition of ileum fatty acids in lyso-phosphatidylcholine (lyso-PC) fraction; Table S4: Percent composition of ileum fatty acids in sphingomyelin (SM) fraction; Table S5: Percent composition of fatty acids in phosphatidylcholine (PC) fraction of mouse ileum; Table S6: Percent composition of ileum fatty acids in phosphatidylserine (PS) fraction; Table S7: Percent composition of ileum fatty acids in phosphatidylinositol (PI) fraction; Table S8: Percent composition of ileum fatty acids in phosphatidylethanolamine (PE) fraction.
