# Supplementary material for: Effect of Lifelong Exposure to Dietary Plant and Marine Sources of n-3 Polyunsaturated Fatty Acids on Morphologic and Gene Expression Biomarkers of Intestinal Health in Early Life
Source: Nutrients. 2024 Mar 1;16(5):719. doi: 10.3390/nu16050719 (PMC10934214; doi:10.3390/nu16050719)
Supplement: Supplementary file 1 [file nutrients-16-00719-s001.zip › nutrients-2884435-supplementary.pdf]

Table S1. Fatty acid composition of modified AIN-93G rodent diets[1].

Fatty Acid (% of total fatty acids)	10% Safflower	3% Menhaden	3% Flaxseed
12:0	0.04	0.08	0.03
14:0	0.24	2.69	0.22
16:0	6.92	10.18	6.52
16:1c9	0.15	4.02	0.13
18:0	2.59	2.87	2.89
18:1c9	15.27	13.86	16.60
18:1c11	0.80	1.48	0.78
18:2n6	72.00	52.04	53.84
18:3n6	0.11	0.16	0.00
18:3n3	0.31	0.87	17.31
18:4n3	0.13	0.90	0.14
20:0	0.36	0.36	0.51
20:1c11	0.22	0.49	0.34
20:2n6	0.05	0.12	0.09
20:3n6	0.00	0.10	0.00
20:4n6	0.00	0.50	0.00
20:3n3	0.00	0.20	0.00
20:5n3	0.05	4.22	0.00
22:0	0.30	0.27	0.28
22:1n9	0.02	0.09	0.02
22:2n6	0.00	0.28	0.00
22:4n6	0.11	0.14	0.00
22:5n3	0.00	0.75	0.00
24:0	0.14	0.02	0.16
22:6n3	0.00	3.10	0.00
24:1	0.17	0.23	0.15
Total %	100	100	100
% Saturated	10.59	16.46	10.61
% Monosaturated	16.63	20.16	18.02
% n-3 polyunsaturated	0.49	10.05	17.45
% n-6 polyunsaturated	72.28	53.33	53.93

[1]Values are means and represent percent of total fatty acids in each diet. Analyses were performed from randomly selected diet pellets in duplicate and fatty acid composition was determined by gas chromatography. Diets: SO [10% safflower oil (D04092701)]; MO [3% menhaden oil + 7% safflower oil (D04092703)]; FX [3% flaxseed oil + 7% safflower oil, (D04092711N)].

Table S2. Lipid concentration (μg/0.1 ileum) in phospholipid classes and PC: PE ratio[1].

Lipid class	SO	MO	FX
Lyso-PC	21.6 ± 7.19	22.4 ± 3.98	20.7 ± 3.03
SM	65.0 ± 18.0	50.0 ± 21.0	50.6 ± 8.63
PC	542 ± 43.2	489 ± 81.2	577 ± 58.4
PS[t]	95.1 ± 27.7	88.4 ± 23.0	102 ± 23.6
PI	140 ± 28.1[b]	110 ± 24.2[a]	148 ± 13.4[b]
PE	399 ± 54.8[b]	319 ± 53.6[a]	390 ± 41.5[b]
PC:PE ratio	1.38 ± 0.24	1.54 ± 0.11	1.48 ± 0.07

[1] Values are means ± S.D., n=6 per dietary group. Lipid concentrations for lyso-PC, SM, PC, PS, PI and PE classes are expressed in μg total fatty acids per 0.1g of ileum. Different letters, in a row, denote significant (*$p \leq 0.05$*) differences between means by one-way analysis of variance followed by Duncan's multiple range test unless stated otherwise. [t] Data set failed to conform to normal distribution and was subjected to Kruskal-Wallis followed by Wilcoxon 2-sample test.

Table S3. Percent composition of ileum fatty acids in lyso-phosphatidylcholine (lyso-PC) fraction[1].

Fatty Acids	SO	MO	FX
12:0	0.00 ± 0.00	0.00 ± 0.00	0.00 ± 0.00
14:0	0.57 ± 0.36	0.68 ± 0.14	0.61 ± 0.31
15:0	0.49 ± 0.27	0.53 ± 0.11	0.55 ± 0.28
16:0	29.1 ± 5.43^{a}	38.4 ± 3.93^{b}	32.2 ± 4.50^{b}
18:0	29.6 ± 3.72	28.4 ± 2.38	31.3 ± 2.39
19:0[t]	1.96 ± 0.97	1.66 ± 1.01	2.25 ± 1.17
20:0	1.44 ± 0.62	1.35 ± 0.39	1.88 ± 0.43
21:0	0.72 ± 0.89	0.16 ± 0.30	0.47 ± 0.53
22:0	2.07 ± 0.62	1.97 ± 0.61	2.60 ± 0.51
23:0	0.48 ± 0.26	0.58 ± 0.19	0.60 ± 0.35
24:0	0.88 ± 0.53^{a}	1.85 ± 0.78^{b}	1.86 ± 0.51^{b}
**Total SFA**	67.3 ± 4.53^{a}	75.6 ± 1.04^{b}	74.3 ± 3.42^{b}
16:1c9[t]	0.00 ± 0.00^{b}	0.60 ± 0.28^{a}	0.00 ± 0.00^{b}
18:1c9	4.46 ± 0.50^{b}	3.73 ± 0.26^{a}	4.84 ± 0.61^{b}
18:1c11	1.27 ± 0.21	1.13 ± 0.19	1.07 ± 0.23
20:1c11	0.81 ± 0.45	0.55 ± 0.10	0.81 ± 0.32
20:3n9	0.00 ± 0.00	0.00 ± 0.00	0.00 ± 0.00
22:1n9[t]	4.33 ± 1.32^{a}	2.38 ± 1.53^{b}	2.94 ± 1.41^{b}
24:1n9	1.23 ± 0.47	1.12 ± 0.46	1.67 ± 0.50
**Total MUFA**	12.1 ± 1.37^{a}	9.51 ± 1.71^{b}	11.3 ± 1.54^{ab}
18:2n6	8.96 ± 2.99^{a}	5.59 ± 0.85^{b}	4.96 ± 0.59^{b}
18:3n6[t]	0.00 ± 0.00^{a}	0.44 ± 0.37^{b}	0.44 ± 0.36^{b}
18:3n3[t]	0.00 ± 0.00^{a}	0.27 ± 0.26^{b}	0.51 ± 0.28^{b}
18:4n3	0.00 ± 0.00	0.21 ± 0.23	0.15 ± 0.24
20:2n6	1.09 ± 0.29^{a}	0.72 ± 0.19^{b}	0.64± 0.17^{b}
20:3n6	1.43 ± 0.60	1.18 ± 0.17	0.89 ± 0.32
20:4n6	4.73 ± 1.38^{a}	2.54 ± 0.45^{b}	2.08 ± 1.00^{b}
20:3n3	0.00 ± 0.00	0.00 ± 0.00	0.00 ± 0.00
20:5n3	2.63 ± 0.76	1.39 ± 1.36	2.62 ± 1.53
22:2n6	0.00 ± 0.00	0.00 ± 0.00	0.00 ± 0.00
22:4n6	0.83 ± 0.33^{a}	0.00 ± 0.00^{b}	0.25 ± 0.28^{b}
22:3n3	0.00 ± 0.00	0.00 ± 0.00	0.00 ± 0.00
22:5n6	0.99 ± 0.48^{a}	0.39 ± 0.39^{b}	0.47 ± 0.37^{b}
22:5n3[t]	0.00 ± 0.00^{a}	0.36 ± 0.10^{b}	0.34 ± 0.39^{b}
22:6n3	0.00 ± 0.00^{a}	1.86 ± 1.17^{b}	1.03 ± 0.63^{b}
**Total PUFA**	20.7 ± 3.77^{a}	14.9 ± 1.75^{b}	14.4 ± 2.12^{b}
**Total n6**	18.0 ± 3.16^{a}	10.9 ± 1.61^{b}	9.74 ± 1.30^{b}
**Total n3**	2.63 ± 0.76^{a}	4.08 ± 0.28^{b}	4.65 ± 1.17^{b}
**n6/n3[t]**	7.12 ± 1.33^{a}	2.67 ± 0.36^{b}	2.23 ± 0.74^{b}

[1] Values are means ± S.D., n=6 per dietary group. Fatty acids are expressed as percent composition of total fatty acid content. Different letters, in a row, denote significant (*p≤0.05*) differences between means by one-way analysis of variance followed by Duncan's multiple range test unless stated otherwise. [t] Data set failed to conform to normal distribution and was subjected to Kruskal-Wallis followed by Wilcoxon 2-sample test.

Table S4. Percent composition of ileum fatty acids in sphingomyelin (SM) fraction[1].

Fatty Acids	SO	MO	FX
12:0[t]	0.00 ± 0.00	0.37 ± 0.46	0.43 ± 0.44
14:0[t]	1.39 ± 1.37	0.95 ± 043	1.02 ± 0.17
15:0[t]	0.71 ± 0.57	0.58 ± 0.21	0.62 ± 0.10
16:0[#]	41.1 ± 9.99	44.1 ± 7.45	47.2 ± 2.88
18:0	17.0 ± 4.41[a]	12.5 ± 2.52[b]	11.5 ± 1.68[b]
19:0[t]	1.52 ± 1.78	0.70 ± 0.12	0.89 ± 0.32
20:0	2.89 ± 0.92	3.56 ± 0.66	4.04 ± 1.07
21:0	0.13 ± 0.14[a]	0.50 ± 0.17[b]	0.40 ± 0.23[b]
22:0	4.07 ± 1.24[a]	5.79 ± 1.21[b]	6.01 ± 1.19[b]
23:0	1.29 ± 0.59	1.68 ± 0.33	1.64 ± 0.25
24:0	2.96 ± 1.01[a]	4.92 ± 1.05[b]	5.17 ± 0.58[b]
**Total SFA**	73.0 ± 5.42	75.6 ± 14.6	78.9 ± 8.88
16:1c9	0.21 ± 0.12	0.53 ± 0.54	0.35 ± 0.33
18:1c9	3.34 ± 1.51	3.07 ± 1.74	2.45 ± 1.07
18:1c11	0.97 ± 0.24	0.90 ± 0.62	0.60 ± 0.26
20:1c11[t]	0.53 ± 0.44[a]	0.07 ± 0.12[b]	0.08 ± 0.13[b]
20:3n9	0.00 ± 0.00	0.00 ± 0.00	0.00 ± 0.00
22:1n9	1.35 ± 0.62	1.09 ± 0.23	1.33 ± 0.19
24:1n9	5.17 ± 1.50[a]	6.74 ± 1.65[ab]	7.95 ± 1.88[b]
**Total MUFA**	11.6 ± 4.43	12.4 ± 4.90	12.8 ± 3.86
18:2n6	8.97 ± 4.35	6.62 ± 5.13	3.65 ± 2.13
18:3n6[t]	0.18 ± 0.22	0.11 ± 0.19	0.16 ± 016
18:3n3[t]	0.15 ± 0.26[b]	0.04 ± 0.07[b]	0.33 ± 0.09[a]
18:4n3	0.00 ± 0.00	0.00 ± 0.00	0.00 ± 0.00
20:2n6	0.53 ± 0.11[b]	0.27 ± 0.30[ab]	0.12 ± 0.22[a]
20:3n6	0.92 ± 0.54	0.94 ± 0.58	0.64 ± 0.23
20:4n6	2.40 ± 0.97[a]	1.22 ± 0.61[b]	0.97 ± 0.36[b]
20:3n3	0.00 ± 0.00	0.00 ± 0.00	0.00 ± 0.00
20:5n3	0.84 ± 0.44	1.10 ± 0.42	1.10 ± 0.12
22:2n6	0.46 ± 0.32	0.40 ± 0.25	0.53 ± 0.20
22:4n6[t]	0.58 ± 0.56[a]	0.06 ± 0.09[b]	0.06 ± 0.09[b]
22:3n3	0.00 ± 0.00	0.00 ± 0.00	0.00 ± 0.00
22:5n6[t]	0.23 ± 0.21	0.13 ± 0.15	0.16 ± 0.25
22:5n3[t]	0.00 ± 0.00	0.13 ± 0.16	0.10 ± 0.17
22:6n3	0.18 ± 0.12[a]	0.96 ± 0.20[c]	0.55 ± 0.06[b]
**Total PUFA**	15.4 ± 4.04[a]	12.0 ± 6.49[ab]	8.37 ± 2.48[b]
**Total n6**	14.3 ± 3.98[b]	9.75 ± 6.34[ab]	6.29 ± 2.25[a]
**Total n3**	1.17 ± 0.58[a]	2.24 ± 0.68[b]	2.08 ± 0.28[b]
**n6/n3**	14.4 ± 7.21[a]	4.58 ± 3.08[b]	2.98 ± 0.78[b]

[1] Values are means ± S.D., n=6 per dietary group. Fatty acids are expressed as percent composition of total fatty acid content. Different letters, in a row, denote significant ($p \leq 0.05$) differences between means by one-way analysis of variance followed by Duncan's multiple range test unless stated otherwise. [#]Analysis of variance of squared-transformed data. [t] Data set failed to conform to normal distribution and was subjected to Kruskal-Wallis followed by Wilcoxon 2-sample test.

Table S5. Percent composition of fatty acids in phosphatidylcholine (PC) fraction of mouse ileum[1].

Fatty Acids	SO	MO	FX
12:0	0.07 ± 0.09	0.08 ± 0.07	0.17 ± 0.10
14:0	0.78 ± 0.50	0.73 ± 0.09	0.80 ± 0.11
15:0	0.32 ± 0.15^{a}	0.38 ± 0.06^{ab}	0.48 ± 0.01^{b}
16:0	23.7 ± 1.30^{b}	26.0 ± 2.29^{a}	23.6 ± 0.68^{b}
18:0	18.2 ± 1.05^{a}	16.3 ± 1.03^{b}	16.9 ± 0.47^{b}
19:0	0.31 ± 0.04	0.26 ± 0.06	0.30 ± 0.05
20:0[t]	0.29 ± 0.03	0.24 ± 0.06	0.28 ± 0.04
21:0	0.00 ± 0.00	0.00 ± 0.00	0.00 ± 0.00
22:0	0.21 ± 0.07	0.17 ± 0.03	0.22 ± 0.03
23:0	0.09 ± 0.02	0.08 ± 0.02	0.10 ± 0.02
24:0	0.24 ± 0.07	0.23 ± 0.07	0.29 ± 0.06
**Total SFA**	44.1 ± 1.34	44.5 ± 3.79	43.1 ± 1.58
16:1c9	0.33 ± 0.12^{a}	0.74 ± 0.17^{b}	0.79 ± 0.07^{b}
18:1c9	7.07 ± 1.68^{b}	7.92 ± 1.43^{b}	10.6 ± 0.64^{a}
18:1c11	1.49 ± 0.21^{a}	2.05 ± 0.22^{b}	1.88 ± 0.07^{b}
20:1c11	0.36 ± 0.08^{b}	0.35 ± 0.06^{b}	0.53 ± 0.07^{a}
20:3n9	0.03 ± 0.04	0.03 ± 0.01	0.06 ± 0.01
22:1n9	0.23 ± 0.11	0.17 ± 0.03	0.22 ± 0.03
24:1n9	0.20 ± 0.07	0.12 ± 0.05	0.15 ± 0.06
**Total MUFA**	9.68 ± 2.26^{b}	11.3 ± 1.97^{b}	14.2 ± 0.94^{a}
18:2n6[t]	28.1 ± 8.78	23.1 ± 1.06	22.9 ± 0.68
18:3n6	0.27 ± 0.07^{b}	0.27 ± 0.04^{b}	0.34 ± 0.02^{a}
18:3n3	0.09 ± 0.04^{b}	0.12 ± 0.03^{b}	0.88 ± 0.09^{a}
18:4n3	0.04 ± 0.02^{b}	0.00 ± 0.00^{a}	0.05 ± 0.01^{b}
20:2n6	1.21 ± 0.37	0.88 ± 0.15	1.09 ± 0.05
20:3n6[#]	2.26 ± 0.90^{a}	3.05 ± 0.17^{b}	2.85 ± 0.15^{b}
20:4n6	11.8 ± 3.41^{a}	9.16 ± 0.97^{b}	8.75 ± 0.68^{b}
20:3n3[t]	00.0 ± 0.00^{b}	0.00 ± 0.00^{b}	0.30 ± 0.02^{a}
20:5n3	0.10 ± 0.05^{a}	2.22 ± 0.20^{c}	1.28 ± 0.13^{b}
22:2n6[t]	0.10 ± 0.04^{a}	0.08 ± 0.01^{ab}	0.12 ± 0.01^{ac}
22:4n6[^]	0.96 ± 0.32^{a}	0.31 ± 0.05^{c}	0.48 ± 0.06^{b}
22:3n3[t]	00.0 ± 0.00^{b}	0.00 ± 0.00^{b}	0.05 ± 0.03^{a}
22:5n6[^]	0.99 ± 0.34^{a}	0.11 ± 0.03^{b}	0.10 ± 0.01^{b}
22:5n3	0.06 ± 0.02^{a}	0.86 ± 0.12^{b}	0.90 ± 0.05^{b}
22:6n3	0.33 ± 0.04^{a}	3.94 ± 0.33^{c}	2.55 ± 0.10^{b}
**Total PUFA**	46.2 ± 3.36^{a}	44.2 ± 1.00^{ab}	42.7 ± 0.47^{b}
**Total n6**	45.5 ± 3.50^{a}	37.0 ± 1.38^{b}	36.6 ± 0.54^{b}
**Total n3**	0.61 ± 0.13^{a}	7.14 ± 0.60^{c}	6.01 ± 0.17^{b}
**n6/n3[^]**	78.3 ± 23.7^{a}	5.22 ± 0.57^{b}	6.09 ± 0.23^{b}

[1] Values are means ± S.D., n=6 per dietary group. Fatty acids are expressed as percent composition of total fatty acid content. Different letters, in a row, denote significant (*p≤0.05*) differences between means by one-way analysis of variance followed by Duncan's multiple range test unless stated otherwise. [^]Analysis of variance of log-transformed data. [#]Analysis of variance of squared-transformed data. [t] Data set failed to conform to normal distribution and was subjected to Kruskal-Wallis followed by Wilcoxon 2-sample test.

Table S6. Percent composition of ileum fatty acids in phosphatidylserine (PS) fraction[1].

Fatty Acids	SO	MO	FX
12:0	0.00 ± 0.00	0.36 ± 0.33	0.49 ± 0.62
14:0[t]	0.09 ± 0.07	0.16 ± 0.12	0.22 ± 0.26
15:0	0.07 ± 0.06	0.11 ± 0.10	0.13 ± 0.14
16:0	3.44 ± 1.22	3.30 ± 0.72	3.39 ± 1.13
18:0	41.9 ± 1.36	42.0 ± 0.19	41.8 ± 1.11
19:0	0.82 ± 0.19	0.76 ± 0.23	0.73 ± 0.21
20:0[t]	0.55 ± 0.09	0.55 ± 0.07	0.62 ± 0.07
21:0	0.00 ± 0.00	0.00 ± 0.00	0.00 ± 0.00
22:0	0.64 ± 0.15	0.71 ± 0.10	0.77 ± 0.23
23:0	0.00 ± 0.00	0.00 ± 0.00	0.00 ± 0.00
24:0[t]	0.44 ± 0.13	0.52 ± 0.31	0.58 ± 0.32
**Total SFA**	47.9 ± 0.74	48.5 ± 0.63	48.7 ± 1.53
16:1c9	0.30 ± 0.22[a]	0.60 ± 0.09[b]	0.76 ± 0.28[b]
18:1c9	8.56 ± 2.57[a]	11.3 ± 1.27[b]	12.9 ± 1.36[b]
18:1c11[t]	0.95 ± 0.09	0.86 ± 0.08	0.91 ± 0.10
20:1c11[t]	0.35 ± 0.18[b]	0.28 ± 0.05[b]	0.42 ± 0.05[a]
20:3n9	0.00 ± 0.00	0.00 ± 0.00	0.00 ± 0.00
22:1n9	0.96 ± 0.27[a]	0.65 ± 0.11[b]	0.81 ± 0.18[ab]
24:1n9[t]	0.30 ± 0.21	0.10 ± 0.11	0.18 ± 0.12
**Total MUFA**	11.4 ± 3.09[a]	13.8 ± 1.26[ab]	16.1 ± 0.80[b]
18:2n6	16.0 ± 3.76	13.3 ± 2.07	13.5 ± 1.85
18:3n6[t]	0.27 ± 0.16	0.33 ± 0.03	0.30 ± 0.17
18:3n3	0.00 ± 0.00[b]	0.08 ± 0.09[b]	0.42 ± 0.06[a]
18:4n3	0.00 ± 0.00	0.00 ± 0.00	0.00 ± 0.00
20:2n6	0.65 ± 0.11[b]	0.48 ± 0.09[a]	0.71 ± 0.07[b]
20:3n6	4.00 ± 1.17[a]	5.26 ± 0.29[b]	5.06 ± 0.56[b]
20:4n6	9.60 ± 1.43[a]	5.89 ± 0.37[b]	5.67 ± 0.66[b]
20:3n3	0.00 ± 0.00	0.00 ± 0.00	0.00 ± 0.00
20:5n3	0.56 ± 0.15[b]	1.14 ± 0.26[a]	0.77 ± 0.41[b]
22:2n6	0.00 ± 0.00	0.00 ± 0.00	0.00 ± 0.00
22:4n6	4.51 ± 0.91[a]	1.19 ± 0.38[b]	1.46 ± 0.49[b]
22:3n3	0.00 ± 0.00	0.00 ± 0.00	0.00 ± 0.00
22:5n6^	3.82 ± 1.06[a]	0.33 ± 0.06[b]	0.39 ± 0.17[b]
22:5n3	0.27 ± 0.23[a]	2.21 ± 0.56[b]	2.23 ± 0.70[b]
22:6n3	0.96 ± 0.13[a]	7.58 ± 2.00[c]	4.64 ± 1.30[b]
**Total PUFA**	40.7 ± 2.72[a]	37.8 ± 1.63[b]	35.2 ± 2.09[b]
**Total n6**	38.9 ± 2.74[a]	26.7 ± 1.74[b]	27.1 ± 0.94[b]
**Total n3**	1.79 ± 0.30[a]	11.0 ± 2.71[c]	8.06 ± 2.34[b]
**n6/n3**	22.3 ± 4.32[a]	2.57 ± 0.72[b]	3.65 ± 1.19[b]

[1] Values are means ± S.D., n=6 per dietary group. Fatty acids are expressed as percent composition of total fatty acid content. Different letters, in a row, denote significant (*p≤0.05*) differences between means by one-way analysis of variance followed by Duncan's multiple range test unless stated otherwise. ^Analysis of variance of log-transformed data. [t] Data set failed to conform to normal distribution and was subjected to Kruskal-Wallis followed by Wilcoxon 2-sample test.

Table S7. Percent composition of ileum fatty acids in phosphatidylinositol (PI) fraction[1].

Fatty Acids	10% Safflower	3% Menhaden	3% Flaxseed
12:0	0.00 ± 0.00	0.00 ± 0.00	0.00 ± 0.00
14:0	0.13 ± 0.12	0.10 ± 0.08	0.10 ± 0.12
15:0	0.07 ± 0.09	0.12 ± 0.12	0.07 ± 0.07
16:0	8.51 ± 1.03	8.80 ± 0.94	7.80 ± 0.54
18:0	36.5 ± 2.49	35.1 ± 2.20	34.6 ± 1.12
19:0	0.64 ± 0.22	0.52 ± 0.13	0.55 ± 0.11
20:0	0.22 ± 0.12[a]	0.30 ± 0.04[ab]	0.36± 0.10[b]
21:0	0.00 ± 0.00	0.00 ± 0.00	0.00 ± 0.00
22:0	0.26 ± 0.17	0.46 ± 0.17	0.49 ± 0.17
23:0	0.00 ± 0.00	0.00 ± 0.00	0.00 ± 0.00
24:0	0.48 ± 0.27	0.58 ± 0.09	0.67 ± 0.10
**Total SFA**	46.8 ± 2.84	46.0 ± 1.69	44.6 ± 0.95
16:1c9	0.37 ± 0.31	0.43 ± 0.13	0.45 ± 0.14
18:1c9	3.77 ± 0.37[b]	3.99 ± 0.67[b]	5.18 ± 0.65[a]
18:1c11[t]	0.93 ± 0.16	1.02 ± 0.24	0.98 ± 0.20
20:1c11	0.14 ± 0.12[a]	0.22 ± 0.16[ab]	0.39 ± 0.17[b]
20:3n9	0.13 ± 0.08	0.16 ± 0.10	0.24 ± 0.14
22:1n9	0.72 ± 0.38	0.56 ± 0.19	0.60 ± 0.15
24:1n9	0.00 ± 0.00	0.00 ± 0.00	0.00 ± 0.00
**Total MUFA**	5.93 ± 0.93[b]	6.23 ± 1.07[b]	7.60 ± 1.07[a]
18:2n6	10.2 ± 2.09	8.50 ± 1.02	8.50 ± 0.97
18:3n6	0.00 ± 0.00	0.00 ± 0.00	0.00 ± 0.00
18:3n3[t]	0.00 ± 0.00[b]	0.00 ± 0.00[b]	0.49 ± 0.10[a]
18:4n3	0.00 ± 0.00	0.00 ± 0.00	0.00 ± 0.00
20:2n6	0.48 ± 0.12	0.40 ± 0.14	0.48 ± 0.08
20:3n6	3.52 ± 0.85[a]	4.78 ± 0.35[b]	4.83 ± 0.53[b]
20:4n6	27.9 ± 2.38[a]	22.1 ± 0.77[b]	23.1 ± 1.15[b]
20:3n3	0.00 ± 0.00	0.00 ± 0.00	0.00 ± 0.00
20:5n3	0.48 ± 0.25[a]	2.24 ± 0.19[c]	1.49 ± 0.38[b]
22:2n6	0.00 ± 0.00	0.00 ± 0.00	0.00 ± 0.00
22:4n6	2.07 ± 0.89[a]	0.97 ± 0.14[b]	1.52 ± 0.33[ab]
22:3n3	0.00 ± 0.00	0.00 ± 0.00	0.00 ± 0.00
22:5n6[t]	1.94 ± 0.89[a]	0.33 ± 0.08[b]	0.20 ± 0.16[b]
22:5n3	0.07 ± 0.08[a]	1.84 ± 0.22[b]	2.15 ± 0.39[b]
22:6n3	0.42 ± 0.35[a]	6.51 ± 0.85[c]	4.77 ± 0.94[b]
**Total PUFA**	47.3 ± 2.69	47.8 ± 1.36	47.8 ± 0.59
**Total n6**	46.1 ± 2.46[a]	37.0 ± 1.52[b]	38.7 ± 1.16[b]
**Total n3**	0.97 ± 0.32[a]	10.6 ± 1.00[c]	8.90 ± 0.90[b]
**n6/n3[t]**	53.0 ± 20.5[a]	3.53 ± 0.45[b]	4.40 ± 0.59[b]

[1] Values are means ± S.D., n=6 per dietary group. Fatty acids are expressed as percent composition of total fatty acid content. Different letters, in a row, denote significant ($p \leq 0.05$) differences between means by one-way analysis of variance followed by Duncan's multiple range test unless stated otherwise. [t] Data set failed to conform to normal distribution and was subjected to Kruskal-Wallis followed by Wilcoxon 2-sample test.

Table S8. Percent composition of ileum fatty acids in phosphatidylethanolamine (PE) fraction[1]

Fatty Acids	SO	MO	FX
12:0^	0.00 ± 0.00	0.11 ± 0.09	0.13 ± 0.14
14:0^	0.12 ± 0.04	0.13 ± 0.03	0.17 ± 0.09
15:0	0.07 ± 0.06	0.10 ± 0.01	0.13 ± 0.06
16:0	5.29 ± 0.37[a]	6.49 ± 0.35[b]	6.02 ± 0.65[b]
18:0	30.8 ± 0.92[a]	28.9 ± 0.63[b]	29.1 ± 1.01[b]
19:0	0.47 ± 0.03	0.40 ± 0.09	0.44 ± 0.07
20:0	0.33 ± 0.02	0.28 ± 0.09	0.35 ± 0.05
21:0	0.00 ± 0.00	0.00 ± 0.00	0.00 ± 0.00
22:0	0.17 ± 0.03	0.17 ± 0.02	0.19 ± 0.03
23:0[t]	0.00 ± 0.00[a]	0.06 ± 0.04[b]	0.04 ± 0.03[b]
24:0	0.19 ± 0.06	0.23 ± 0.05	0.23 ± 0.04
**Total SFA**	37.5 ± 0.77	36.9 ± 0.66	36.8 ± 1.26
16:1c9	0.36 ± 0.17[a]	0.54 ± 0.14[b]	0.56 ± 0.09[b]
18:1c9	6.30 ± 0.60[b]	6.68 ± 0.75[b]	8.69 ± 0.81[a]
18:1c11	0.99 ± 0.10[a]	1.17 ± 0.10[b]	1.20 ± 0.19[b]
20:1c11	0.33 ± 0.03	0.28 ± 0.08	0.36 ± 0.06
20:3n9	0.09 ± 0.05	0.08 ± 0.03	0.11 ± 0.03
22:1n9	0.28 ± 0.13	0.19 ± 0.10	0.42 ± 0.25
24:1n9	0.02 ± 0.03[a]	0.07 ± 0.01[b]	0.09 ± 0.05[b]
**Total MUFA**	8.27 ± 0.80[b]	8.92 ± 1.05[b]	11.3 ± 0.94[a]
18:2n6[t]	16.3 ± 6.54	11.1 ± 0.82	11.6 ± 0.67
18:3n6	0.21 ± 0.10	0.15 ± 0.01	0.18 ± 0.06
18:3n3	0.22 ± 0.07[b]	0.20 ± 0.14[b]	0.87 ± 0.17[a]
18:4n3	0.12 ± 0.09	0.18 ± 0.17	0.26 ± 0.13
20:2n6	0.64 ± 0.10[a]	0.36 ± 0.05[c]	0.51 ± 0.03[b]
20:3n6[#]	1.96 ± 0.87[a]	2.56 ± 0.16[ab]	2.70 ± 0.17[b]
20:4n6	24.0 ± 3.20[a]	17.4 ± 0.79[b]	18.9 ± 1.54[b]
20:3n3[t]	0.00 ± 0.00[b]	0.00 ± 0.00[b]	0.13 ± 0.03[a]
20:5n3	0.14 ± 0.06[a]	3.20 ± 0.13[c]	2.01 ± 0.34[b]
22:2n6	0.08 ± 0.06	0.03 ± 0.02	0.09 ± 0.06
22:4n6^	4.11 ± 1.12[a]	1.21 ± 0.15[c]	1.96 ± 0.18[b]
22:3n3	0.00 ± 0.00	0.00 ± 0.00	0.00 ± 0.00
22:5n6^	4.79 ± 1.43[a]	0.36 ± 0.09[b]	0.29 ± 0.03[b]
22:5n3	0.19 ± 0.04[a]	2.50 ± 0.20[b]	2.92 ± 0.20[c]
22:6n3	1.43 ± 0.16[a]	14.8 ± 0.70[c]	9.33 ± 0.25[b]
**Total PUFA**	54.3 ± 0.94[b]	54.2 ± 1.17[b]	51.9 ± 1.44[a]
**Total n6**	52.1 ± 0.95[a]	33.2 ± 1.05[c]	36.3 ± 1.28[b]
**Total n3**	2.11 ± 0.15[a]	20.9 ± 0.75[c]	15.5 ± 0.76[b]
**n6/n3^**	24.8 ± 1.80[a]	1.59 ± 0.08[c]	2.34 ± 0.14[b]

[1] Values are means ± S.D., n=6 per dietary group. Fatty acids are expressed as percent composition of total fatty acid content. Different letters, in a row, denote significant ($p \le 0.05$) differences between means by one-way analysis of variance followed by Duncan's multiple range test unless stated otherwise. ^Analysis of variance of log-transformed data. [#]Analysis of variance of squared-transformed data. [t] Data set failed to conform to normal distribution and was subjected to Kruskal-Wallis followed by Wilcoxon 2-sample test.
